# Distinct and Shared Roles of β-Arrestin-1 and β-Arrestin-2 on the Regulation of C3a Receptor Signaling in Human Mast Cells

**DOI:** 10.1371/journal.pone.0019585

**Published:** 2011-05-12

**Authors:** Arpana Vibhuti, Kshitij Gupta, Hariharan Subramanian, Qiang Guo, Hydar Ali

**Affiliations:** Department of Pathology, School of Dental Medicine, University of Pennsylvania, Philadelphia, Pennsylvania, United States of America; University of Oldenburg, Germany

## Abstract

**Background:**

The complement component C3a induces degranulation in human mast cells via the activation of cell surface G protein coupled receptors (GPCR; C3aR). For most GPCRs, agonist-induced receptor phosphorylation leads to the recruitment of β-arrestin-1/β-arrestin-2; resulting in receptor desensitization and internalization. Activation of GPCRs also leads to ERK1/2 phosphorylation via two temporally distinct pathways; an early response that reflects G protein activation and a delayed response that is G protein independent but requires β-arrestins. The role of β-arrestins on C3aR activation/regulation in human mast cells, however, remains unknown.

**Methodology/Principal Findings:**

We utilized lentivirus short hairpin (sh)RNA to stably knockdown the expression of β-arrestin-1 and β-arrrestin-2 in human mast cell lines, HMC-1 and LAD2 that endogenously expresses C3aR. Silencing β-arrestin-2 attenuated C3aR desensitization, blocked agonist-induced receptor internalization and rendered the cells responsive to C3a for enhanced NF-κB activity as well as chemokine generation. By contrast, silencing β-arrestin-1 had no effect on these responses but resulted in a significant decrease in C3a-induced mast cell degranulation. In shRNA control cells, C3a caused a transient ERK1/2 phosphorylation, which peaked at 5 min but disappeared by 10 min. Knockdown of β-arrestin-1, β-arrestin-2 or both enhanced the early response to C3a and rendered the cells responsive for ERK1/2 phosphorylation at later time points (10–30 min). Treatment of cells with pertussis toxin almost completely blocked both early and delayed C3a-induced ERK1/2 phosphorylation in β-arrestin1/2 knockdown cells.

**Conclusion/Significance:**

This study demonstrates distinct roles for β-arrestins-1 and β-arrestins-2 on C3aR desensitization, internalization, degranulation, NF-κB activation and chemokine generation in human mast cells. It also shows that both β-arrestin-1 and β-arrestin-2 play a novel and shared role in inhibiting G protein-dependent ERK1/2 phosphorylation. These findings reveal a new level of complexity for C3aR regulation by β-arrestins in human mast cells.

## Introduction

The anaphylatoxin C3a is generated following bacterial infection and from IgE/FcεRI stimulated human mast cells [1]. Accordingly, C3a has been proposed to play critical roles in innate immunity and allergic diseases such as asthma [2], [3], [4]. C3a activates its cell surface G protein coupled receptor (GPCR; C3aR) to induce chemotaxis in human mast cell line (HMC-1) and degranulation in human skin mast cells, peripheral blood CD34^+^ cell-derived mast cells and a differentiated mast cell line, LAD2 [1], [5], [6], [7], [8]. C3a induces mast cell degranulation via the activation of phospholipase Cβ and mobilization of intracellular Ca^2+^ [7], [9]. However, the mechanism(s) involved in regulation of C3aR signaling in mast cells remain poorly understood.

It is well established that for most GPCRs, receptor phosphorylation by G protein coupled receptor kinases (GRKs) and the subsequent recruitment of β-arrestin provides an important mechanism for their desensitization and internalization [10]. Two isoforms of β-arrestins, (β-arrestin-1 and β-arrestin-2) are known and each can differentially regulate GPCR desensitization and internalization. Thus, for protease activated receptor-1 (PAR-1) only β-arrestin-1 is capable for receptor desensitization but receptor internalization is independent of either β-arrestins [11]. By contrast, both isoforms of β-arrestins can promote desensitization of β2-adrenergic receptors (βAR2) and angiotensin II type 1A receptor (AT_1_AR) [12]. Although, only β-arrestin-2 promotes internalization of βAR2 both isoforms are required for the internalization of AT_1_AR. We have previously shown that in transfected rat basophilic leukemia (RBL-2H3) cells, C3aR associates with β-arrestin-2 following agonist stimulation [9]. However, the roles of β-arrestin-1 and β-arrestin-2 on C3aR desensitization and internalization have not been determined.

Previous studies with transfected RBL-2H3 cells showed that phosphorylation-deficient chemoattractant/chemokine receptors that do not associate with β-arrestins respond to ligands for more sustained Ca^2+^ mobilization and degranulation when compared with cells expressing wild-type receptors [9], [13], [14], [15], [16]. These findings are consistent with the view that β-arrestins play an important role in desensitization. By contrast, activation of the chemokine receptor CXCR1 in human neutrophils leads to receptor internalization and complex formation between β-arrestin-2 and Src kinases, (Hck and c-Fgr) which translocate to secretory granules to promote degranulation [17]. β-arrestin-2 also forms a complex with Ral-GDP dissociation stimulator (Ral-GDS) in the cytoplasm of human neutrophils [18]. Furthermore, activation of fMLP receptor results in the translocation of the complex to the plasma membrane. This is followed by the release of Ral-GDS from β-arrestin and the activation of Ral resulting in actin cytoskeleton rearrangement presumably leading to degranulation. The roles of β-arrestins on C3a-induced mast cell degranulation, however, remain unknown.

In addition to receptor desensitization, internalization and degranulation, β-arrestins modulate the activity of the transcription factor, NF-κB. Witherow et al., [19], using a yeast two-hybrid screen, first demonstrated that the inhibitor of NF-κB, IκBα binds to β-arrestin-1. Furthermore, both β-arrestin-1 and 2 interact with IκBα in transfected cells. However, siRNA-mediated knockdown studies indicated that β-arrestin-1 but not β-arrestin-2 inhibits TNF-α-induced NF-κB activation. By contrast, Gao et al., [20] showed that β-arrestin-2, but not β-arrestin-1 interacts with IκBα to inhibit NF-κB activation. Studies with primary leukocytes from β-arrestin-2 knockout mice showed that this adapter molecule is involved in the internalization of the chemokine receptor, CXCR2 [21]. Furthermore, *in vivo* studies showed that β-arrestin-2 deletion promotes tumor growth and angiogenesis and these responses are associated with enhanced chemokine generation [22]. Other studies have shown that both β-arrestins promote NF-κB activation following the activation GPCRs [23], [24]. The roles of β-arrestins on C3a-induced NF-κB activation and chemokine production in mast cells have not been determined.

The mitogen-activated protein kinases (MAPKs), extracellular signal-regulated kinases 1 and 2 (ERK1/2) play important roles in a variety of cellular responses and have been studied for a variety of GPCRs. Activation of GPCRs in transfected cell lines induce ERK1/2 phosphorylation via two temporally distinct pathways; an early response that reflects G protein activation and an delayed response that is G protein independent but requires the formation of signaling complexes involving Src/ERK with β-arrestin [25], [26]. Although C3a induces ERK1/2 phosphorylation in mast cells [7], [9], [27], whether or not β-arrestin-1 and β-arrestin-2 regulate this response remains unknown.

The goal of the present study was to determine the roles of β-arrestins on the regulation of C3aR signaling in human mast cells. To achieve this goal, we utilized lentivirus shRNA to stably knockdown the expression of β-arrestin-1 and β-arrestin-2 in human mast cells (HMC-1 and LAD2). Using these systems, we report unexpected findings regarding distinct roles of these adapter molecules on C3aR desensitization, internalization, degranulation, NF-κB activation and chemokine generation. Furthermore, we provide first demonstration that both β-arrestins acts as novel inhibitors of C3a-induced G-protein-mediated ERK1/2 phosphorylation in human mast cells.

## Results

### Stable knockdown of β-arrestin-1 and β-arrestin-2 in a human mast cell, HMC-1

**Figure 1:**
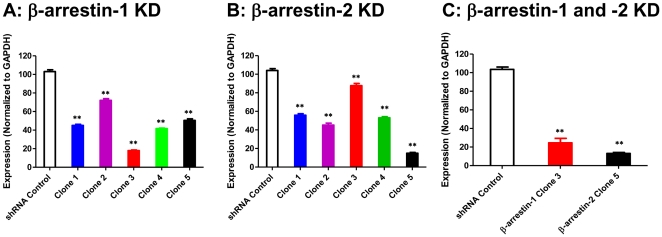
Stable knockdown of β-arrestin-1 and β-arrestin-2 in human mast cells. HMC-1 cells were stably transduced with scrambled shRNA control lentivirus or different clones of shRNA lentivirus targeted against β-arrestin-1 and β-arrestin -2 (Panels A and B). For double knockdown HMC-1 cell were transduced with shRNA lentivirus, Clone 3 of β-arrestin-1 and Clone 5 of β-arrestin-2 (C). Quantitative PCR was employed to assess β-arrestin-1 or -2 mRNA levels. Results are expressed as a ratio of β-arrestin to GAPDH mRNA levels. Data represent the mean ± SEM from three independent experiments. Statistical significance was determined by one way ANOVA. ** indicates p<0.001.

To determine the role of β-arrestins on the regulation of C3aR signaling in mast cells, we used the Mission shRNA lentivirus system to stably knockdown the expression of β-arrestin-1 and β-arrestin-2 in a human mast cell line; HMC-1 cells. Cells were transduced with 5 different shRNA constructs targeting different regions of β-arrestin-1 and β-arrestin-2. For control, we used a scrambled shRNA construct. After transduction and selection with puromycin, quantitative real-time PCR was performed to determine the extent of β-arrestin knockdown. As shown in Fig. 1A and B, all five β-arrestin shRNA constructs decreased the expression of β-arrestin-1 and β-arrestin-2 to variable levels. Clone 3 (TRCN0000230149) for β-arrestin-1 and clone 5 (TRCN0000159482) for β-arrestin-2 showed >80% decrease in mRNA. We therefore, used these clones to generate double knockdown (both β-arrestin-1 and β-arrestin-2) in HMC-1 cells. As shown in Fig. 1C, we were able to generate HMC-1 cells with ∼80% knockdown of both genes. These cells were used in subsequent studies described below.

### β-arrestin-2, but not β-arrestin-1, is required for C3aR desensitization and internalization

**Figure 2:**
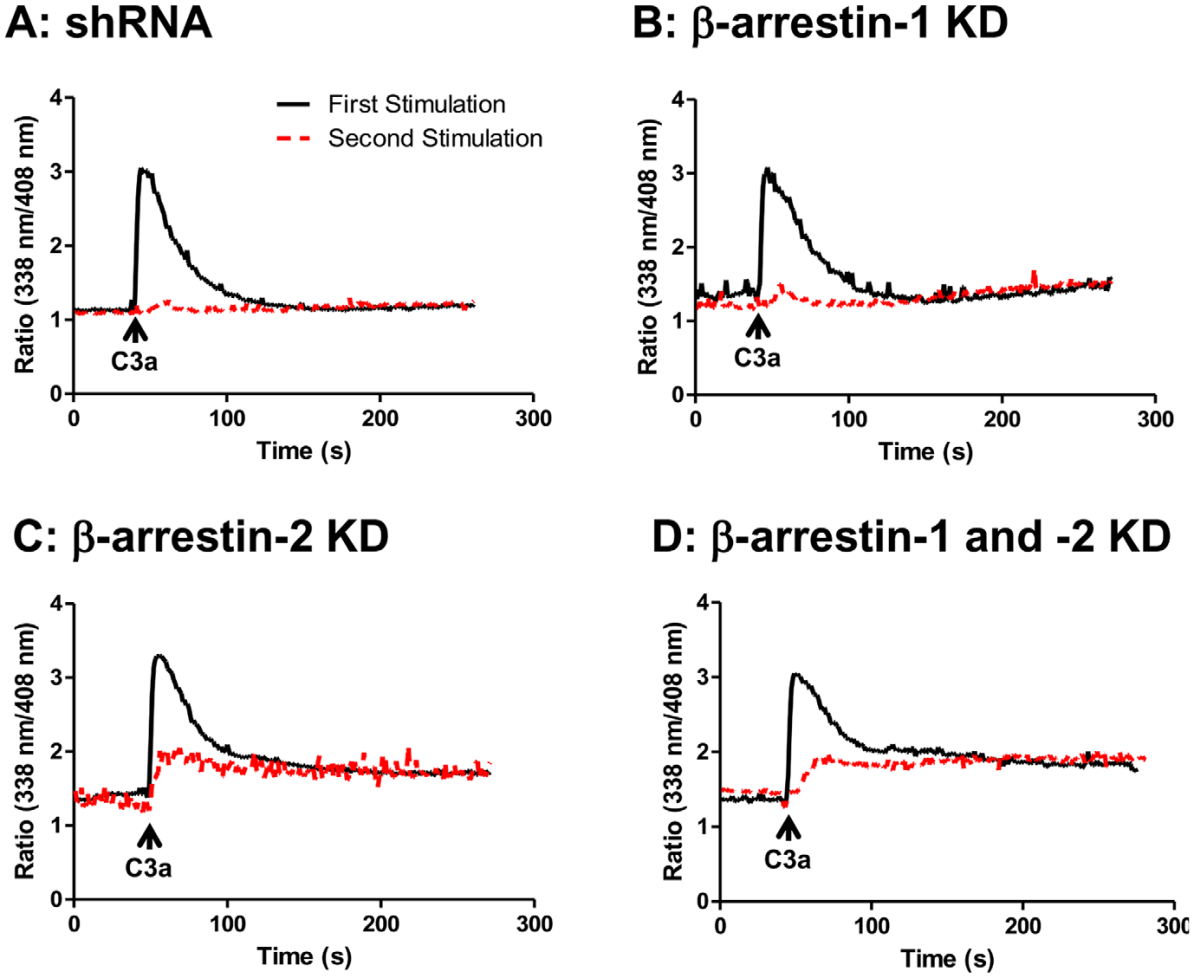
Knockdown of β-arrestin-2, but not β-arrestin-1, attenuates C3aR desensitization. (A), shRNA control, (B) β-arrestin-1 KD (knockdown), (C) β-arrestin-2 KD and (D) double β-arrestin-1 and 2 KD cells were loaded with Indo-1(1 µM), stimulated with C3a (100 nM) for 5 min and intracellular Ca^+2^ mobilization was determined (black solid lines). The cells were immediately washed three times with ice-cold buffer, resuspended in warm buffer and exposed to a second stimulation of C3a (100 nM) and intracellular Ca^2+^ mobilization was again determined (red broken lines). Data shown are representative of three similar experiments.

Intracellular Ca^2+^ mobilization provides a rapid, sensitive and real-time assay to measure receptor desensitization [28]. We have previously shown that receptors that undergo desensitization respond to agonists with an initial Ca^2+^ spike, which decays rapidly and reaches baseline within ∼2–3 min [28]. By contrast, phosphorylation-deficient receptors that do not associate with β-arrestin respond to agonist for a similar initial Ca^2+^ spike, followed by a sustained response that remains elevated for an extended period of time [29], [30]. We therefore, used Ca^2+^ mobilization as an assay to determine the effects of β-arrestin-1 and β-arrestin-2 knockdowns on C3aR desensitization. As shown in Fig. 2A and B, C3a caused a rapid increase in Ca^2+^ mobilization in shRNA control and β-arrestin-1 knockdown cells. By contrast, in β-arrestin-2 knockdown C3a caused a similar initial spike but subsequent response was sustained (Fig. 2C). Furthermore, deletion of both β-arrestins resulted in a Ca^2+^ response similar to that observed in β-arrestin-2 knockdown cells (Fig. 2C and D). These findings suggest that β-arrestin-2, but not β-arrestin-1, mediates desensitization of C3aR.

GPCRs that undergo desensitization display reduced responsiveness to a second stimulation with the same agonist [28]. To test further the effects of β-arrestins on desensitization, shRNA control or knockdown cells were exposed to C3a and washed twice before re-exposure to the same concentration of C3a. In shRNA control and β-arrestin-1 knockdown cells, there was little or no response to second C3a stimulation. Interestingly, β-arrestin-2 knockdown cells responded to re-exposure to C3a for Ca^2+^ mobilization (Fig. 2C). Notably, for initial Ca^2+^ mobilization and desensitization, double knockdown cells responded similarly to β-arrestin-2 knockdown cells (Fig. 2D).

**Figure 3:**
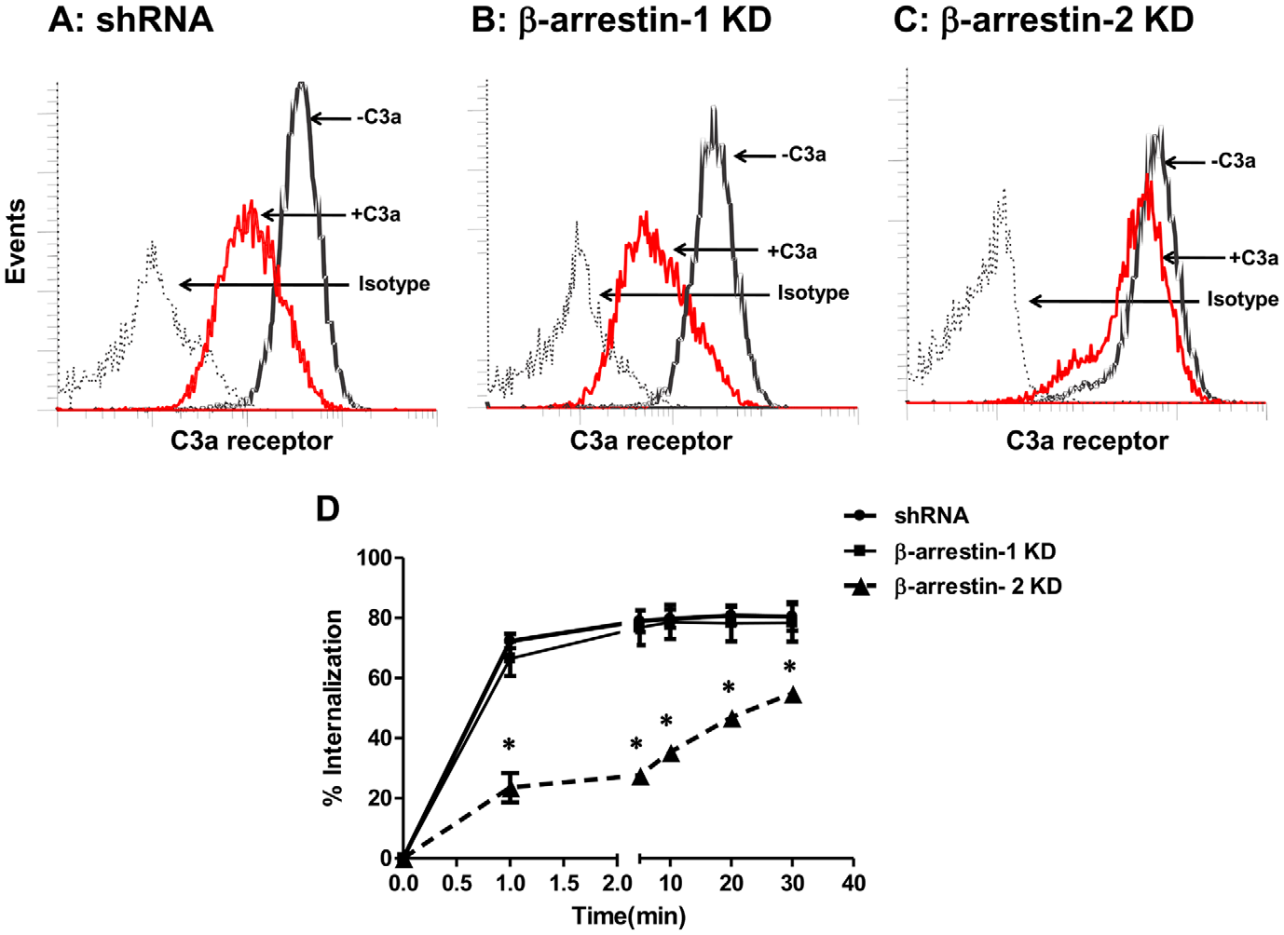
Knockdown of β-arrestin-2, but not β-arrestin-1, inhibits agonist-induced C3aR internalization. (A) shRNA control HMC-1 cells (B) β-arrestin-1 KD and (C) β-arrestin-2 KD cells were exposed to buffer (−C3a) or C3a (100 nM) for 5 min. Cells were washed with ice-cold FACS buffer, incubated with a mouse anti-C3aR antibody or an isotype control antibody followed by PE-labeled donkey anti-mouse IgG antibody and analyzed by flow cytometry. Representative histograms showing cell surface C3aR expression in (A) shRNA control, (B) β-arrestin-1 KD and (C) β-arrestin-2 KD cells are shown. (D) shRNA control, β-arrestin-1 KD and β-arrestin-2 KD cells were exposed to C3a for different time periods and receptor internalization was determined as described above. Internalization is expressed as the percentage loss of C3aR following exposure to C3a. Data represent the mean ± SEM from three experiments. Statistical significance was determined by two way ANOVA with Bonferroni's post test. * indicates p<0.05.

To investigate the role of β-arrestins on agonist-induced C3aR internalization, shRNA control, β-arrestin-1 and β-arrestin-2 knockdown cells were exposed to buffer or C3a and receptor internalization was determined by flow cytometry. In shRNA control cells, C3a caused a robust internalization of its receptors (Fig. 3A). In β-arrestin-1 knockdown cells, there was no marked difference in the extent of receptor internalization (Fig. 3A, B). Interestingly, internalization of C3aR was substantially reduced in β-arrestin-2 knockdown cells (Fig. 3C and D). These findings clearly demonstrate that β-arrestin-2, but not β-arrestin-1, is involved in C3aR desensitization and internalization in human mast cell line, HMC-1.

### β-arrestin-1, but not β-arrestin-2, promotes C3a-induced mast cell degranulation

**Figure 4:**
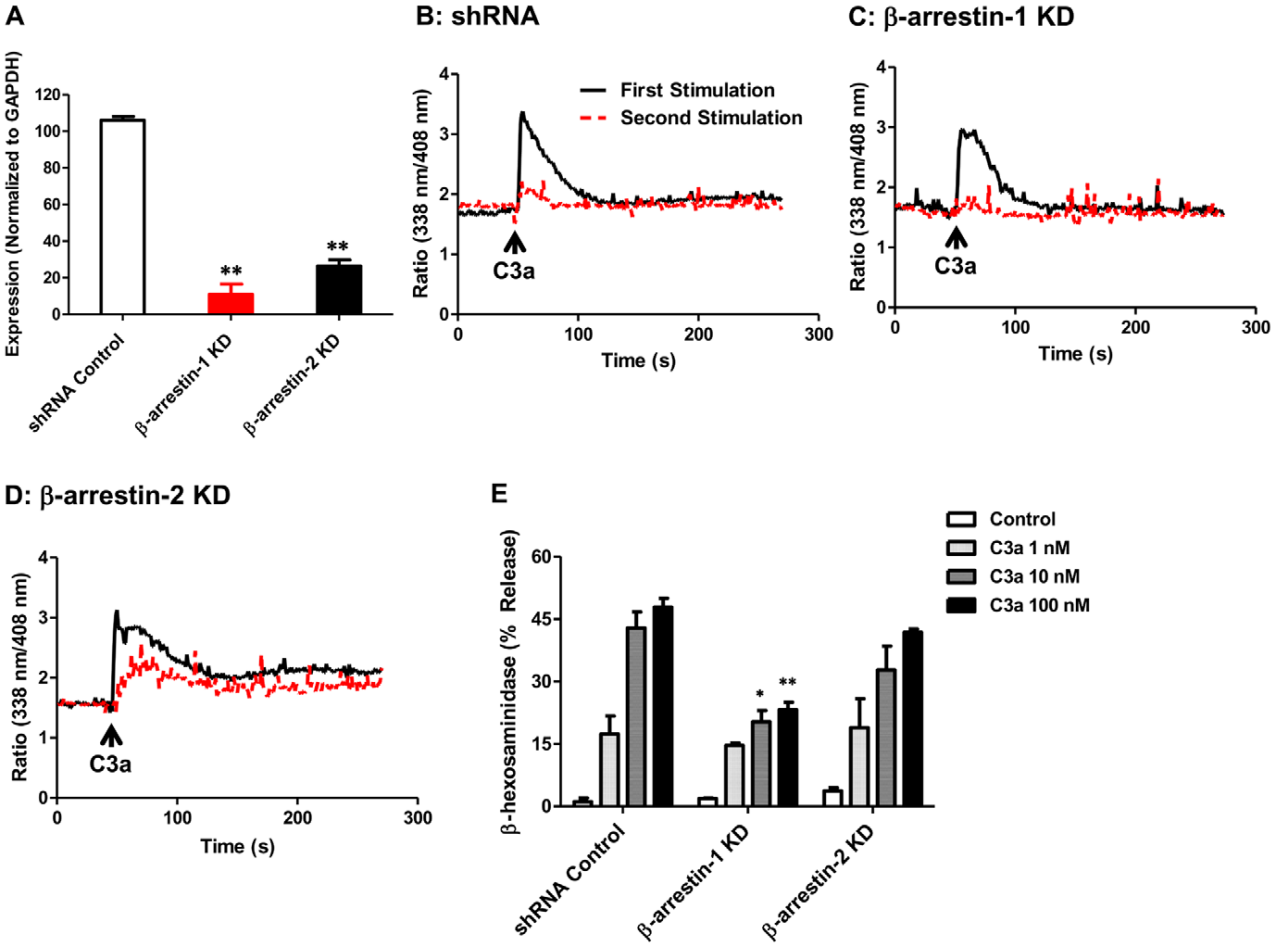
Knockdown of β-arrestin-1, but not β-arrestin-2, inhibits C3a-induced degranulation in LAD2 mast cells. (A) LAD2 cells were transduced with shRNA control, β-arrestin-1, and β-arrestin-2 lentivirus. After puromycin selection, quantitative PCR was employed to assess the β-arrestin-1 or β-arrestin-2 mRNA level and results are expressed as a ratio of β-arrestin to GAPDH mRNA levels. (B, C, D) A representative desensitization experiment in shRNA control, β-arrestin-1 and β-arrestin-2 knockdown LAD2 cells is shown. (E) shRNA control, β-arrestin-1 and -2 KD LAD2 mast cells were stimulated with different concentrations of C3a and percent degranulation (β-hexosaminidase release) was determined. Data represent the mean ± SEM from three independent experiments. Statistical significance was determined by two way ANOVA with Bonferroni's post test. * indicates p<0.05 and ** indicates p<0.001.

Our next goal was to determine the roles of β-arrestin-1 and β-arrestin-2 on C3a-induced mast cell degranulation. We could not use HMC-1 cells for these studies because this immature mast cell line has little or no capacity to degranulate. LAD2 mast cells express C3aR and responds to ligand for Ca^2+^ mobilization and degranulation [7]. We therefore knocked down the expression of β-arrestin-1 and β-arrestin-2 in LAD2 cells. As in HMC-1 cells, lentiviral shRNA induced ∼80% knockdown of the β-arrestin-1 and β-arrestin-2 in LAD2 mast cells (Fig. 4A). Furthermore, consistent with the findings in HMC-1 cells, β-arrestin-1 knockdown in LAD2 cells had little or no effect on C3a-induced Ca^2+^ mobilization (Fig. 4B and C) while β-arrestin-2 silencing resulted in a more sustained Ca^2+^ mobilization and loss of desensitization (Fig. 4D). Surprisingly, however, knockdown of β-arrestin-2 had no effect on C3a-induced mast cell degranulation but the absence of β-arrestin-1 resulted in a significantly decreased degranulation response (Fig. 4E).

### β-arrestin-2, but not β-arrestin-1, inhibits C3a-induced NF-κB activation and chemokine CCL4 generation

**Figure 5:**
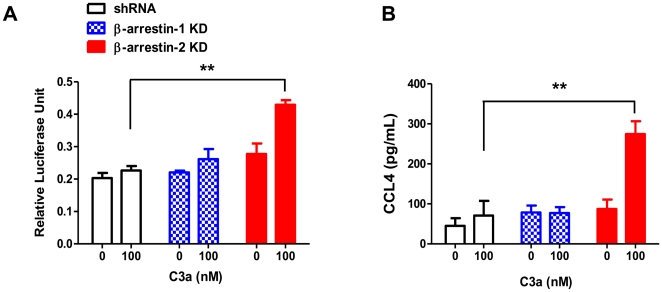
Knockdown of β-arrestin-2, but not β-arrestin-1, enhanced C3a-induced NF-κB activation and chemokine CCL4 generation. shRNA control, β-arrestin-1 KD or β-arrestin-2 KD HMC-1 cells were transiently transfected with NF-κB luciferase reporter gene construct. (A) Cells were stimulated with C3a (100 nM for 6 hr) and NF-κB-dependent transcriptional activity was determined by luciferase activity assay. (B) Control or β-arrestin KD cells were stimulated with C3a (100 nM for 6 hr) and CCL4 production was determined from the supernatant by ELISA. Data shown are mean ± SEM of three experiments performed in triplicate. Statistical significance was determined by two way ANOVA with Bonferroni's post test. ** indicates p<0.001.

β-arrestin-1 and β-arrestin-2 bind to IκBα to inhibit GPCR-induced NF-κB activity in transfected cell lines [19], [20]. We therefore sought to determine the roles of these adapter molecules on C3a-induced NF-κB luciferase activity in human mast cells. We used HMC-1 cells for these studies because they are more amenable to transfection than LAD2 cells. C3a did not induce NF-κB luciferase activity in shRNA control or β-arrestin-1 silenced HMC-1 cells (Fig. 5A). By contrast, β-arrestin-2 knockdown cells showed a significant enhancement in C3a-induced NF-κB luciferase activity as compared to shRNA control cells. Given that NF-κB plays an important role in the generation of proinflammatory cytokines, we tested the effects of β-arrestin-1 and β-arrestin-2 knockdown in C3a-induced chemokine CCL4 production. Consistent with NF-κB activation, C3a induced CCL4 only in β-arrestin-2 silenced cells (Fig. 5B).

### β-arrestin-1 and β-arrestin-2 inhibit C3a-induced ERK1/2 phosphorylation

**Figure 6:**
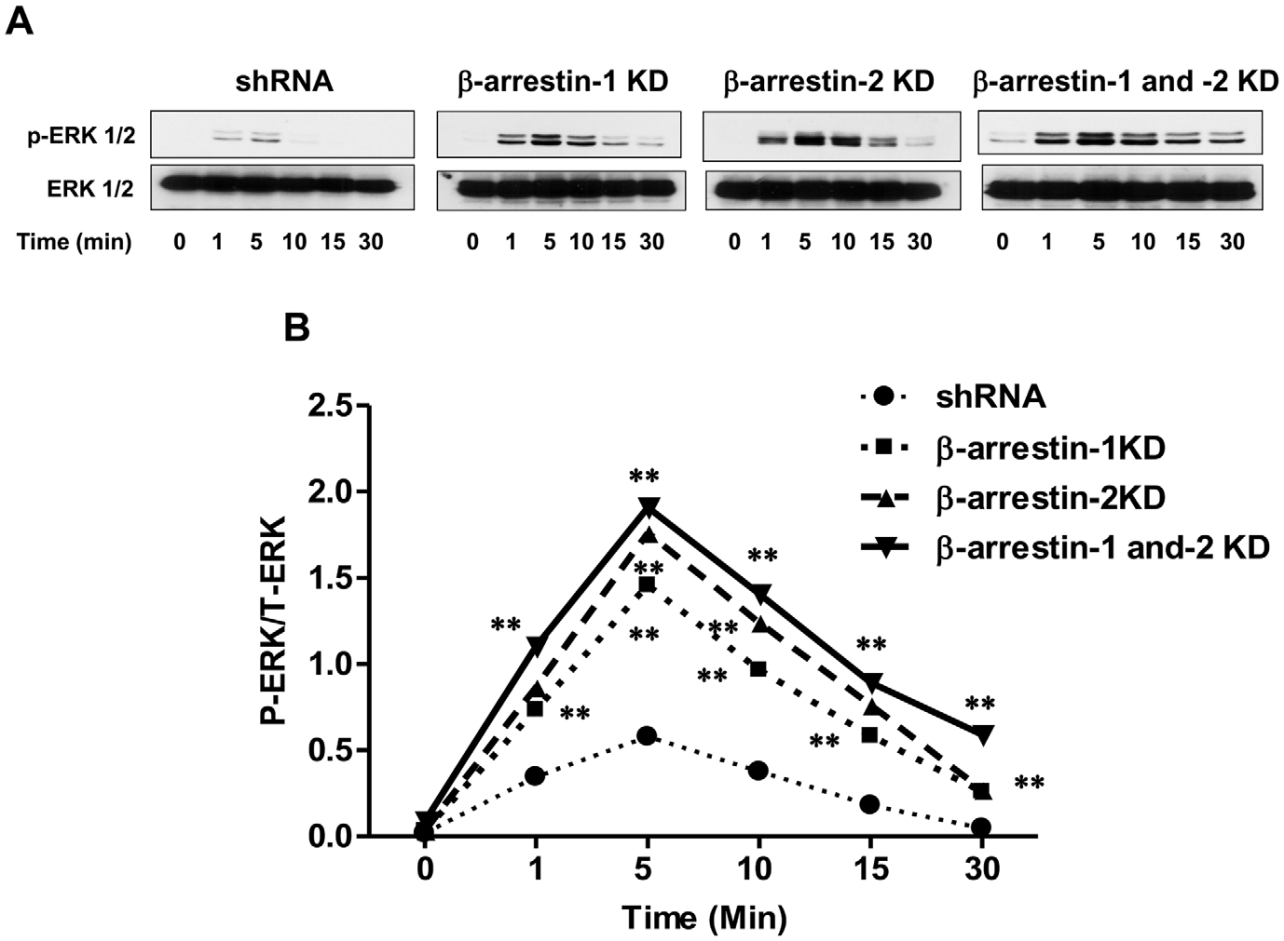
C3a-induced ERK1/2 phosphorylation is enhanced in β-arrestin-1, β-arrestin-2 and double KD cells. shRNA control or β-arrestin KD HMC-1 cells (1×10^6^/ml) were exposed to C3a (100 nM) for 1, 5 and 10, 15 and 30 min. Cell lysates were separated on SDS-PAGE and blots were probed with anti-phospho-ERK1/2 antibody followed by anti-rabbit IgG-HRP. The blots were then stripped and reprobed with anti-ERK1/2 antibody followed by anti-rabbit IgG-HRP. Immunoreactive band were visualized by SuperSignal West Femto maximum sensitivity substrate. (A) Representative immunoblots from three similar experiments are shown. (B) ERK1/2 phosphorylation was quantified using Image J as shown in the line graph. Data represent the mean ± SEM from three independent experiments. Statistical significance was determined by two way ANOVA with Bonferroni's post test. ** indicates p<0.001.

Activation of GPCRs leads to ERK1/2 phosphorylation via two temporally distinct pathways; an early response that reflects G protein activation and a delayed response that is G protein independent but requires β-arrestins [31]. We therefore investigated the effects of silencing the expression of β-arrestin-1, β-arrestin-2 or both on the time course of C3a-induced ERK1/2 phosphorylation in HMC-1 cells. In shRNA control cells, C3a caused a transient ERK1/2 phosphorylation that peaked between 1–5 min and returned to basal thereafter (Fig. 6A). Surprisingly, silencing β-arrestin-1 or β-arrestin-2 expression enhanced the magnitude of this early response and rendered the cells responsive to C3a for ERK1/2 phosphorylation even at later time points (10–30 min). Furthermore, in double knockdown cells, C3a-induced ERK1/2 phosphorylation was greater in magnitude than single β-arrestin knockdown cells (Fig 6A and 6B).

**Figure 7:**
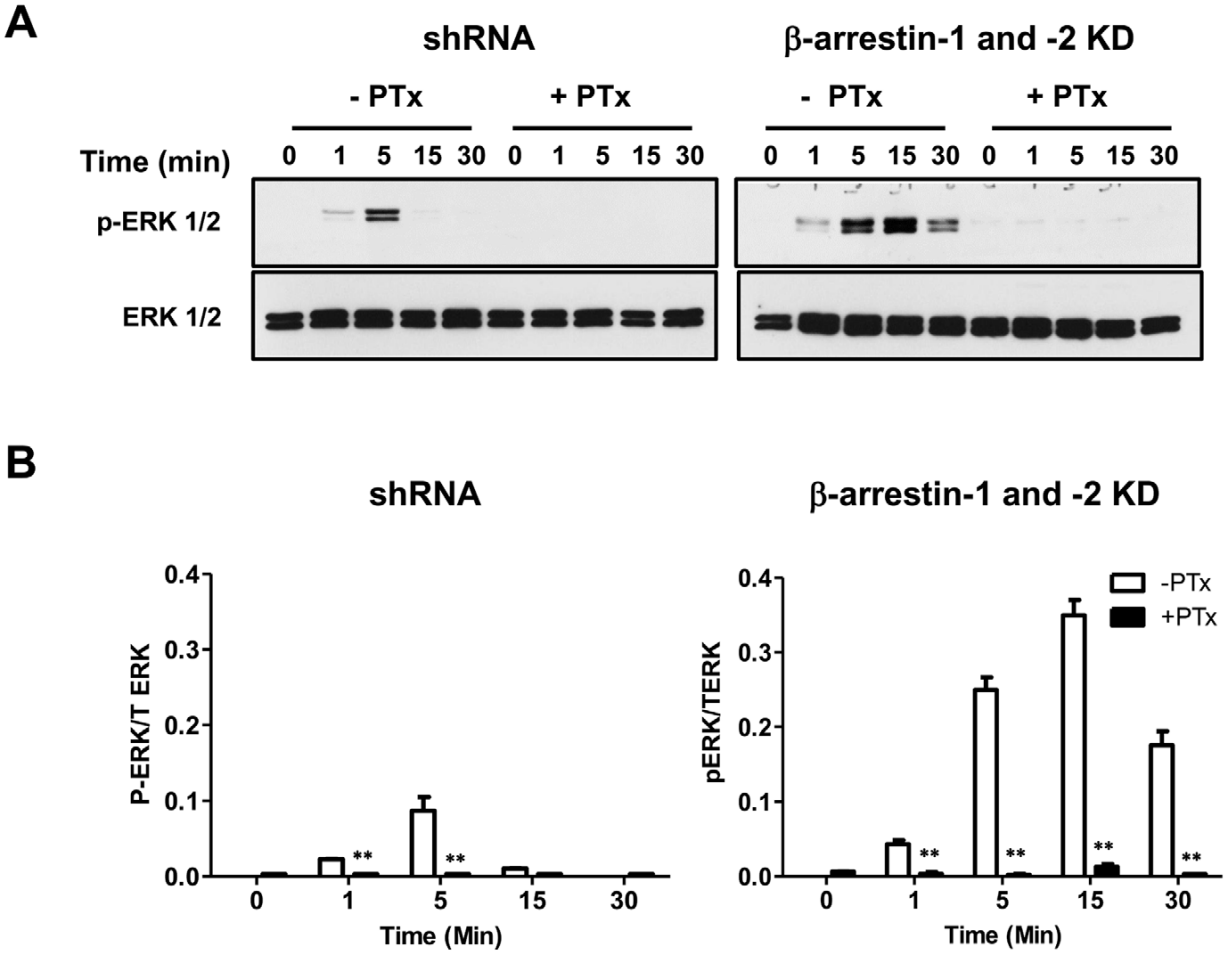
Enhanced C3a-induced ERK1/2 phosphorylation in β-arrestin KD cells mast is mediated via a G protein-dependent pathway. shRNA control and double β-arrestin KD cells were pretreated with vehicle or Pertussis toxin (PTx; 100 ng/ml, 16 hr). Cell were then washed twice in serum free medium and stimulated with C3a (100 nM) for 1, 5 and 10, 15 and 30 min and ERK1/2 phosphorylation were determined. (A) Representative immunoblots from three similar experiments are shown. (B). ERK1/2 phosphorylation was quantified using Image J as shown in the bar graph. Data represent the mean ± SEM from three independent experiments. Statistical significance was determined by two way ANOVA with Bonferroni's post test. ** indicates p<0.001.

To determine if the delayed C3a-induced ERK1/2 phosphorylation in β-arrestin knockdown cells is mediated via a G protein-dependent pathway, shRNA control and β-arrestin-1 and β-arrestin-2 double knockdown cells were exposed to pertussis toxin and the effects of C3a on ERK1/2 phosphorylation was determined. As shown in Fig. 7A and 7B both early and delayed responses were almost completely inhibited in pertussis toxin treated cells.

## Discussion

β-arrestins are well known for their roles in GPCR desensitization and internalization. They also modulate downstream signaling pathways such as those for ERK and NF-κB independent of receptor desensitization. Most previous studies on GPCR regulation have been performed using mouse embryonic fibroblasts (MEFs) derived from β-arrestin null mice [11], transfected cell lines overexpressing β-arrestins or siRNA-mediated β-arrestin knockdown in HEK293 cells [32], [33], [34], [35]. For the present study, we utilized lentivirus shRNA to stably knockdown the expression of β-arrestin-1 and β-arrestin-2 in human mast cell lines, HMC-1 cells and LAD2 cells that endogenously express C3aR. Using this approach, we have uncovered distinct roles of β-arrestin-1 and β-arrestin-2 on C3aR desensitization, internalization, degranulation, NF-κB activation and chemokine generation. Furthermore, we provided the first demonstration that β-arrestin-1 and β-arrestin-2 act as novel inhibitors of C3a-induced G protein-dependent ERK1/2 phosphorylation in human mast cells.

**Figure 8:**
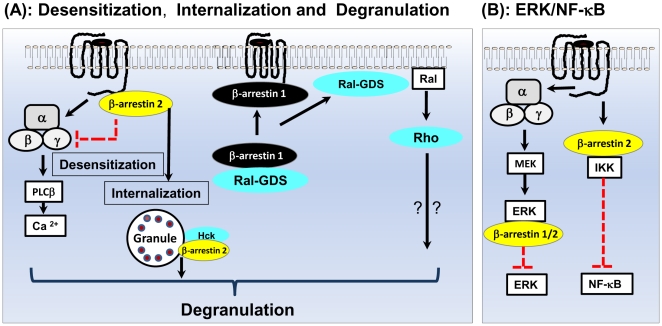
Model for the Regulation of C3aR signaling in human mast cells by β-arrestin-1 and β-arrestin-2. (A): β-arrestin-2 causes desensitization and internalization of C3aR but both β-arrestins promote G protein-independent signaling for degranulation via the activation of Hck and/or Ral-GDS-mediated signaling pathways [17], [18]. (B) β-arrestin-2 mediates inhibition of C3a-induced NF-κB activity but both β-arrestins block C3a-induced G-protein-mediated ERK1/2 phosphorylation. Red dotted lines denote inhibition.

Previous studies indicated that β-arrestin-2 either inhibits or promotes chemoattractant/chemokine induced degranulation. Thus, in response to agonist stimulation wild-type chemoattractant receptors associate with β-arrestin-2 in transfected RBL-2H3 cells but phosphorylation-deficient mutants do not [9], [14], [16], [30]. Furthermore, agonist-induced Ca^2+^ mobilization and degranulation are enhanced in cells expressing phosphorylation-deficient receptors when compared to wild-type receptors. These findings are consistent with the notion that receptor phosphorylation and β-arrestin-2 participate in receptor desensitization. By contrast, Barlic et al., [17] showed that agonist induced phosphorylation of the chemokine receptor CXCR1 leads to β-arrestin-2-mediated receptor internalization and the formation of β-arrestin-2-Hck complex, which migrates to secretory granules initiating the process of degranulation. The finding in the present study that enhanced Ca^2+^ response in the absence of β-arrestin-2 did not promote greater degranulation provides a possible explanation for the previously published conflicting data for the role of β-arrestin-2 on degranulation. It suggests that β-arrestin-2 plays a dual role on GPCR-induced degranulation; inhibition via desensitization and activation via its association with Hck. Thus, the inability of enhanced Ca^2+^ response to promote greater degranulation in β-arrestin-2 knockdown cells probably reflects the loss of β-arrestin-2-mediated Hck signaling (see Model in Fig. 8A).

An interesting finding of the present study was that while knockdown of β-arrestin-1 had no effect on C3aR desensitization (as measured by Ca^2+^ mobilization) or receptor internalization its absence resulted in a substantial inhibition of C3a-induced mast cell degranulation. Because β-arrestins 1 does not participate in C3aR internalization, C3a is unlikely to promote Hck-β-arrestin-1 interaction. Our studies with confocal microscopy in live cells indicated that C3a causes translocation of β-arrestin-1 to the plasma membrane (data not shown). Furthermore, β-arrestin 1 forms a complex with Ral-GDS in the cytoplasm of human neutrophils [18]. This raises the interesting possibility that upon C3aR activation, β-arrestin-1/Ral GDS complex translocates to the plasma membrane to promote degranulation and that knockdown of β-arrestin-1 leads to attenuated response due to the absence of this complex (see Model in Fig. 8A). Whether this or other mechanism(s) participate on the effect of β-arrestin-1 on C3a-induced mast cell degranulation remains to be determined.

β-arrestins have been shown to promote or inhibit NF-κB activity depending on the cell type and receptors utilized [14], [19], [20], [23], [24]. Our results clearly demonstrate that β-arrestin-2, but not β-arrestin-1, inhibits C3a-induced NF-κB activation and chemokine generation. Gao et al., [20] recently observed similar differences between β-arrestin-1 and β-arrestin-2 in cytokine production in Hela cells and THP-1 monocytes. This difference was thought to reflect a reduced ability of β-arrestin-1 to form a complex with the inhibitory IκBα when compared to β-arrestin-2. In the present study, we showed that while β-arrestin-1 does not participate in agonist-induced C3aR internalization, β-arrestin-2 is essential for this response. It is therefore possible that internalized C3aR-β-arrestin-2 complex interacts with IκBα to keep NF-κB inactive and that depletion of β-arrestin-2 removes this inhibitory constraint to allow NF-κB activation and chemokine generation (Fig. 8B). It is also possible that enhanced signaling as manifested by a more sustained Ca^2+^ mobilization in β-arrestin-2 knockdown cells results in greater NF-κB activation and chemokine generation.

An interesting finding of the present study was that silencing β-arrestin-1 and β-arrestin-2 enhanced early ERK1/2 phosphorylation in response to C3a and also rendered the cells responsive to C3a at later time points (15–30 min). This finding is in direct contrast to situations with many other GPCRs, where β-arrestins are required for delayed ERK phosphorylation [31]. One possible interpretation of our finding is that knockdown β-arrestins attenuate C3aR desensitization leading to more sustained ERK1/2 phosphorylation. This explanation is, however, unlikely as β-arrestin-1 knockdown, which had no impact on C3aR desensitization, rendered the cells responsive to C3a for ERK1/2 phosphorylation. Ahn et al. [33] recently demonstrated that β-arrestin-2 inhibits angiotensin II type 1A-mediated ERK1/2 activation in HEK293 cells and silencing β-arrestin-1 expression enhances this response by blocking β-arrestin-2 mediated inhibition. This type of reciprocal regulation is unlikely for C3a-induced response in mast cells as knockdown of both β-arrestins either individually or together enhanced C3a-induced ERK1/2 phosphorylation.

It is noteworthy that *Drosophila* genome encodes a single β-arrestin, Kurtz (Krz), which controls olfaction, behavior, sensitivity to osmotic stress, and is essential for survival of the fly [36], [37], [38]. Tipping et al., [39] recently showed that Krz directly binds to and sequesters an inactive form of ERK, thus preventing its activation by the upstream kinase, MEK. We have shown that pertussis toxin completely blocks the transient C3a-induced ERK1/2 phosphorylation in shRNA control cells as well as the sustained response in β-arrestin-1 and β-arrestin-2 double knockdown cells. This finding suggests that C3a causes ERK1/2 phosphorylation via a GPCR-mediated pathway and that arrestins inhibit this response by forming a direct complex with ERK and preventing it activation by MEK (Fig. 8B).

In summary, we demonstrated distinct roles for β-arrestins-1 and β-arrestins-2 on C3aR desensitization, internalization, degranulation, NF-κB activation and chemokine generation in human mast cells. Most importantly, we provided the first demonstration that both β-arrestin-1 and β-arrestin-2 act as novel inhibitors of C3a-induced G-protein-mediated ERK1/2 phosphorylation in human mast cells. In addition to C3aR, human mast cells express a large number of other GPCRs, FcεRI, toll-like receptors and IL-33 receptor T1/ST2 [40], [41], [42], [43], [44]. Given that β-arrestins regulate GPCR and non-GPCR signaling [45], [46], it is likely that they regulate other receptor/signaling pathways in human mast cells. Our future studies will focus on the receptor specificity of human mast cell regulation by β-arrestins.

## Materials and Methods

### Materials

Mission shRNA bacterial glycerol stocks for β-arrestins were purchased from Sigma Life Sciences (St. Louis, MO). Indo-1 AM was from Molecular Probes (Eugene, OR). All tissue culture reagents were purchased from Invitrogen (Gaithersburg, MD). Anti-human C3aR was obtained from Santa Cruz Biotechnology (Santa Cruz, CA), PE-labeled donkey anti-mouse IgG was purchased from eBioscience (San Diego, CA). All recombinant human cytokines were purchased from Peprotech (Rocky Hill, NJ). Rabbit anti-ERK1 and anti-phospho-ERK1/2 antibodies were purchased from Cell Signaling (Beverly, MA). SuperSignal® West Femto Maximum Sensitivity Substrate and HRP labeled Goat anti-rabbit IgG were from Thermo Scientific (Rockford, IL). Purified C3a was obtained from Advanced Research Technologies (San Diego, CA). CCL4 ELISA kit was purchased from R&D Systems (Minneapolis, MN).

### Mast cell culture

HMC-1 cells were cultured in Iscove's modified Dulbecco's medium (IMDM) supplemented with 10% FCS, glutamine (2 mM), penicillin (100 IU/mL) and streptomycin (100 µg/mL) [47]. LAD2 cells were maintained in complete StemPro-34 medium supplemented with 100 ng/mL rhSCF [48].

### Lentivirus and stable transduction of shRNAs in mast cells

The following β-arrestin-1 and -2 targeted shRNAs in Lentiviral construct plasmid were purchased from Sigma-Aldrich (St. Louis, MO): β-arrestin-1 (NM_004041) Clone 1 TRCN0000230148, Clone 2 TRCN0000230147, Clone 3 TRCN0000230149, Clone 4 TRCN0000230150, Clone 5 TRCN0000219075; β-arrestin-2 (NM_004313) Clone 1 TRCN0000165387, Clone 2 TRCN0000164794, Clone 3 TRCN0000159332, Clone 4 TRCN0000161834, Clone 5 TRCN0000159482 and control non-target vector SHC002. Cell transduction was conducted by mixing 1.5 ml of virus with 3.5 ml of HMC-1 or LAD-2 cells (5×10^6^). For the double knockdown of β-arrestin-1 and -2, 1.5 ml of each virus of specific clones were transduced in 2 ml of HMC-1 or LAD-2 cells (5×10^6^). Eight hr post-infection, medium was changed to virus-free complete medium, and antibiotic (puromycin; 2 µg/ml Sigma-Aldrich) selection was initiated 16 h later. Cells were analyzed for β-arrestin knockdown one week after initiation of puromycin selection.

### Real-Time PCR

Total RNA was extracted from 4×10^6^ of cells using TRIZOL, treated with DNase I and subsequently purified for genomic DNA contamination with RNeasy mini Kit (Qiagen) according to the manufacture's instruction. cDNA was synthesized from genomic DNA-free RNA using the cDNA synthesis kit from GE Healthcare. Gene expression was analyzed using real time PCR with Taqman® Fast Universal PCR Master Mix on a Taqman 7500 Fast Real-Time PCR System (Applied Biosystems, Foster City, CA). Taqman hGAPDH, β-arrestin-1 and β-arrestin-2 primers were used for real time PCR to analyze the knockdown efficiency. The amplification conditions were as follows: initial denaturation at 95°C for 20 sec, followed by 40 cycles of amplification: 95°C for 3 sec, 60°C for 30 sec. Analysis was performed according to ΔΔ-Ct method. The results were expressed as β-arrestin-1 or -2/*GAPDH* ratio.

### C3a Receptor desensitization

Receptor desensitization assay based on Ca^2+^ mobilization was determined as described previously [49]. Briefly, 1×10^6^ HMC-1 or 0.25×10^6^ LAD-2 cells were washed twice with buffer (119 mM NaCl, 5 mM KCl, 25 mM HEPES, 5.6 mM Glucose, 0.4 mM MgCl2, 1 mM CaCl_2_) containing 1 mg/ml BSA and incubated with 1 µM of Indo-1 for 30 min in dark. Cells were then washed and resuspended in 1.5 ml of the same buffer and time course of Ca^2+^ mobilization (0–5 min) was determined using Hitachi F-2500 Fluoro spectrophotometer (San Jose, CA) with an excitation wavelength of 355 nM and an emission wavelength of 410 nM [9]. For desensitization assay, cells were removed from the cuvette, washed twice and Ca^2+^ mobilization to a subsequent exposure of C3a (100 nM) was determined.

### Degranulation Assay

LAD-2 cells (1.2×10^4^) were seeded into 96-well plates in a total volume of 50 µl of buffer containing 1 mg/ml BSA and exposed to different concentrations of C3a (1, 10 and 100 nM). For total β-hexosaminidase release, control cells were lysed in 50 µl of 0.1% Triton X-100. Aliquots (20 µl) of supernatants or cell lysates were incubated with 20 µl of 1 mM p-nitrophenyl-N-acetyl-β-D-glucosamine for 1.5 hour at 37°C. The reaction was stopped by adding 250 µl of a 0.1 M Na_2_CO_3_/0.1 M NaHCO_3_ buffer and absorbance measured at 405 nm [49].

### Receptor Internalization

ShRNA control and β-arrestin knockdown HMC-1 cells (2.5×10^5^) were stimulated with or without C3a (100 nM) at 37°C. Cells were washed twice and resuspended in 50 µl of ice-cold FACS buffer (PBS containing 2% FBS). C3aR antibody or isotype control (2 µl) was added and the cells were incubated on ice for 1 h. Cells were washed twice and re-suspended in 48.5 µl of ice-cold FACS buffer. Phycoerythrin (PE)-labeled donkey anti-mouse (1.5 µl) was added and incubated on ice for 1 h. Cells were washed twice with cold FACS buffer and fixed in 250 µl of 2% formaldehyde. Receptor internalization was quantified as the loss of cell-surface receptors, as analyzed on a BD LSR II flow cytometer (BD Biosciences).

### ERK1/2 Phosphorylation

ShRNA control and β-arrestin knockdown HMC-1 cells were serum starved overnight. The following day, cells were washed twice and resuspended in serum free IMDM medium at a concentration of 1×10^6^/ml and stimulated C3a (100 nM ) for different time points. Three-fold volume of ice-cold PBS containing 1 mM sodium orthovanadate was added to stop the reaction. Total cell lysate was prepared with RIPA buffer (150 mM NaCl, 1.0% NP-40, 0.5% Sodium-deoxycholate, 0.10% SDS, 50 mM Tris [pH 8.0], 5 mM EDTA, 10 mM NaF, 10 mM Na-pyrophosphate and protease inhibitor cocktail) and subsequently analyzed by Western blot using rabbit polyclonal antibodies for phospho-p44/42 MAPK (pERK1/2) and p44/42 MAPK (ERK1/2).

### NF-κB luciferase reporter activity

ShRNA control and β-arrestin knockdown HMC-1 cells (3×10^6^) were seeded in 12-well plates. The following day, cells were co-transfected with NF-κB luciferase reporter gene construct (pNF-κB-LUC and p-Renilla Stratagene, Santaclara, CA) (in a 10∶1 ratio) using Lipofectamine 2000 reagent (Invitrogen, Carlsbad, CA) in serum-free IMDM medium as per manufacturer protocol. Six hour post-transfection, medium was replaced with IMDM containing 10% FBS. After 18 hr of incubation in complete medium, cells were re-plated and stimulated in the presence or absence of 100 nM C3a for 6 hr. Cells were then harvested, washed in ice-cold PBS and finally lysed in Promega passive lysis buffer (Dual Luciferase assay kit; Promega, Madison, WI). NF-κB luciferase activity was measured using Turner biosystem 20/20 Luminometer (Promega, Madison, WI). Results expressed have been normalized to Renilla.

### CCL4 chemokine release assay

Chemokine release assay was performed as previously described [27]. HMC-1 shRNA control, β-arrestin-1 and β-arrestin-2 knockdown cells (0.2×10^6^ cells) were stimulated with 100 nM C3a for 6 hours. CCL4 chemokine levels were quantified by sandwich ELISA according to the manufacturer's protocol.

### Data analysis

The results are expressed as ± S.E.M for the values obtained from experiment. GraphPad Prism software (Graph Pad, Version 5.0 San Diego, CA) was used to analyze data for statistical significance. The statistical significance was determined by one-way analysis of variance (ANOVA) with Dunnett's multiple comparison post hoc test, and two way ANOVA with Bonferroni's post test.
